# Hygienic-dietary recommendations for major depression treatment: Study protocol of a randomized controlled trial

**DOI:** 10.1186/1471-244X-12-201

**Published:** 2012-11-16

**Authors:** Mauro Garcia-Toro, Miquel Roca, Saray Monzón, Margalida Vives, Bárbara Oliván, Enric Vicens, Joan Salva, Margalida Gili

**Affiliations:** 1 Institut Universitari d’Investigació en Ciències de la Salut (IUNICS). Universitat de les Illes Balears (UIB). Red de Investigación en Actividades Preventivas y Promoción de la Salud (redIAPP), Palma de Mallorca, Spain; 2 Facultad de Ciencias de la Salud, Universidad de San Jorge, Red de Investigación en Actividades Preventivas y Promoción de la Salud (redIAPP), Zaragoza, Spain; 3 Departamento de Psiquiatría, Parc Sanitari San Joan de Déu, Red de Investigación en Actividades Preventivas y Promoción de la Salud (redIAPP), Barcelona, Spain; 4 Departamento de Psiquiatría, Hospital Son Espases, Palma de Mallorca, Spain

**Keywords:** Depression, Hygienic-dietary treatment, Lifestyle recommendations

## Abstract

**Background:**

Depression is a highly prevalent and disabling mental disorder with an incidence rate which appears to be increasing in the developed world. This fact seems to be at least partially related to lifestyle factors. Some hygienic-dietary measures have shown their efficacy as a coadjuvant of standard treatment. However, their effectiveness has not yet been proved enough in usual clinical practice.

**Methods:**

Multicenter, randomized, controlled, two arm-parallel, clinical trial involving 300 patients over 18 years old with a diagnosis of Major Depression. Major depression will be diagnosed by means of the Mini-International Neuropsychiatric Interview. The Beck Depression Inventory total score at the end of the study will constitute the main efficacy outcome. Quality of Life and Social and Health Care Services Consumption Scales will be also administered. Patients will be assessed at three different occasions: baseline, 6-month follow-up and 12-month follow-up.

**Discussion:**

We expect the patients in the active lifestyle recommendations group to experience a greater improvement in their depressive symptoms and quality of life with lower socio-sanitary costs.

**Trial registration:**

ISRCTN73931675

## Background

Depression is a highly prevalent mental disorder in our society, with 10 to 20% of the population experiencing a major depressive disorder/depressive episode during their lives [1-4]. From the health care system perspective, some studies confirm that 25-35% of patients consuming Primary Care services have a psychiatric condition, with over 80% of these patients presenting depression or anxiety disorders [5]. It is well known that General Practitioners (GPs) only refer approximately 5-10% of the psychiatric patients detected in Primary Care to Mental Health Services [6]. Despite this low referral rate, Mental Health Services in occidental countries are currently congested due to the high prevalence of minor psychiatric problems and this situation is very likely to worsen in the future [7]. Thus, it is of great interest to propose cost-effective alternatives for depression treatment in Primary Care settings which could improve efficacy to standard treatment while tolerability and security levels are sustained.

An essential issue regarding this alarming increment of the incidence and prevalence rates of depression is the fact that it seems to be occurring mainly in occidental countries [8,9]. Hence, it is necessary to rapidly clarify which variables could explain this trend in order to modify them [9,10]. It is presumed that lifestyle in occidental countries contribute to this increase in depression rates although specific factors involved have not been yet identified [8-10]. No important findings from the point of view of genetics or neurobiological factors have been published within the last decades, with the exception of a probable increase in alcohol and other drugs consumption which in any case is not sufficient to fully explain this trend [11]. Consequently, it has been more frequently attributed to environmental changes in our lifestyle especially those related to stress, nonconformity, competitiveness and loss of social relationships [12]. Besides the great importance of these described factors, in recent times other variables have been submitted which might possibly influence this increase [13]. In this sense, it has been suggested that occidental lifestyle implies changes in diet, hours of sleep, sun exposure and physical activity, all of which could play a role as a mediator variable for depression onset [8,9,12,13]. More and more people are decreasing their physical activity level and following a less healthy diet [14,15]. Moreover, there is a tendency towards a reduction of sleep hours and time of sunlight exposure [16,17]. It is well known that these changes impact the physiology of the brain which can lead to an increment in the vulnerability to depression [8]. Accordingly, it is not surprising that adjusting sleep hours, physical exercise, diet and sunlight exposure have been recommended separately by different research groups as a complementary or adjuvant treatment to pharmacotherapy and psychotherapy [18-20]. However, there is little investigation about the usefulness of GPs giving these simple hygienic-dietary measures as a recommendation jointly to antidepressant treatment in usual clinical practice conditions [9,12].

We conducted a preliminary study [9,13] where 80 patients undertaking standard treatment with antidepressants were randomly assigned to two groups. Patients in the intervention group received written recommendations about physical exercise, Mediterranean diet, sunlight exposure and sleep control. Obtained results were encouraging [9].

## Methods

### Hypothesis and objectives

The first hypothesis of the present study is that patients assigned to the intervention group receiving specific hygienic-dietary recommendations will show a significantly greater improvement in clinical measures of depression in comparison to control group patients. The second hypothesis is that significant differences in health costs will be found between both groups, with patients in the intervention group showing lower expenses. Therefore, the main objective is to evaluate the effectiveness and efficiency of giving specific hygienic-dietary recommendations to depressive patients in Primary Care settings. Secondary objectives are to evaluate changes in direct and indirect costs, to specify a profile of patients who benefit from this intervention and to identify the most frequent inconveniences for patients leading to non-adherence to these recommendations.

The present study is a randomized controlled clinical trial under “real world” conditions. Patients will be allocated to one of these two conditions:

1 Treatment as usual prescribed by their GP plus specified hygienic-dietary recommendations (intervention group)

2 Treatment as usual prescribed by GP plus unspecified hygienic-dietary recommendations (control group)

The study design is shown in Table 1.

**Table 1:** STUDY SCHEDULE

	***“TRIAGE”***	***BASELINE***	***SIXTH MONTH***	***TWELFTH MONTH***
**Criteria compliance evaluation**	**X**			
**Discussion of the study information leaflet**	**X**			
**Signed Consent Form**	**X**			
**MINI Interview**		**X**		
**Sociodemographic questionnaire**		**X**		
**Clinic questionnaire**		**X**	**X**	**X**
**Physical parameters**				
**BP**		**X**		**X**
**HR**		**X**		**X**
**BMI**		**X**		**X**
**WAIST CIRCUMFERENCE**		**X**		**X**
**Blood Test**		**X**		**X**
**Scales**				
**BDI-21**		**X**	**X**	**X**
**STAI**		**X**	**X**	**X**
**EQ-5D**		**X**	**X**	**X**
**CSRI**		**X**	**X**	**X**
**Delivery of Hygienic-dietetic recommendations**		**X**		

### Study sample

Patients meeting the following inclusion criteria will be recruited: individuals aged 18 or older with a diagnosis of Major Depressive Disorder as stated by the DSM-IV, presenting mild to moderate depressive symptoms (as measured by BDI) of at least two months of duration, and having sufficient physical and cognitive aptitudes to understand and give written informed consent.

Among the exclusion criteria are the comorbidity with other medical conditions which would affect the Central Nervous System (CNS), other severe psychiatric disorders (except for anxiety disorders or personality disorders), or other non-controlled severe medical diseases, infectious or degenerative which would interfere with affective symptomatology or the adherence to the hygienic-dietary recommendations. Moreover, patients with delusions or hallucinations which are congruent or non-congruent with mood during study and patients with an important risk for suicide will also be excluded.

Recruitment of patients will take place in Primary Care Health Centers within the Balearic Islands, Catalonia and Aragon (Spain).

### Sample size and randomization

Statistical analysis will be based on head-to-head comparisons between the group receiving the hygienic-dietary recommendations and the “treatment as usual” group. Based on previous research, we expect that the mean difference between both groups in the main variable (BDI Total score) will be of at least 3 points (SD=6). Assuming an alpha risk of 0.01 and two tails, with a study power of 90% the corresponding sample size is 119 patients per group. With an estimated withdrawal rate of 20%, the sample size will require approximately 150 patients in each group. Thus, the total sample size of the study will be 300 patients.

Randomization will take place for each patient once participation is agreed to and the written informed consent signed. Every GP participating in this study will be provided with identically sealed envelopes codified with a numerical code that identifies the contents. These codes will be kept by the head of the research team. Half of the envelopes will contain the specific hygienic-dietary recommendations under consideration while the other half will contain “control” unspecified hygienic-dietary recommendations. In all cases, these recommendations will be written on a sheet of paper. The GP will randomly choose an envelope and ask the patient to follow the recommendations written inside.

### Intervention

Patients in the intervention group will receive the following specific hygienic-dietary recommendations:

IN ORDER TO SPEED UP THE AMELIORATION OF YOUR DEPRESSIVE SYMPTOMS, WE ENCOURAGE YOU TO FOLLOW THESE HYGIENIC-DIETARY RECOMMENDATIONS:

1. Go to bed only when you feel sleepy and never before 11 p.m. Use your bedroom only for sleeping and sexual relations. Do not read, watch TV or stay inside the bedroom with any other purpose during the day. If you are unable to fall asleep within 15–20 minutes after going into bed, get up and get involved in an activity until you feel sleepy enough to go to bed again. Get up early, even if you have not slept properly. Do not get up later than 9 a.m. in any case. Do not lie down during the day and avoid taking a nap if you have not slept well the previous night.

2. Walk for a minimum of one hour every day, with a pace with which you do not feel breathless or are unable to speak. If you think you suffer any medical condition that would advise against this recommendation, please ask your doctor. Use comfortable trainers and wash after exercising by having a shower or a bath.

3. Expose yourself to environmental sun light, taking appropriated cautions to avoid sunburn or sunstroke (sunscreen, hat, etc.)

4. Try to follow a healthy and well-balanced diet. Eat at regular times, avoiding snacks between meals, especially sweets and sweetened drinks. Consume legumes and fish at least three times per week. Also, eat fruits, cereals, nuts and dried food, and other vegetables daily.

Patients in the control group will receive an identical envelope containing a sheet with the following unspecified hygienic-dietary recommendations:

IN ORDER TO SPEED UP THE AMELIORATION OF YOUR DEPRESSIVE SYMPTOMS, WE ENCOURAGE YOU TO FOLLOW THESE HYGIENIC-DIETARY RECOMMENDATIONS:

1. Sleep the hours that you feel your body needs.

2. Adapt the pace of daily physical activity to meet your needs best.

3. If exposed to sunlight take precautions to avoid sunburn or sunstroke (sunscreen, hat, etc.).

4. Try to eat a healthy and balanced diet.

### Outcomes and instruments

The main outcome in this study is symptom severity as measured by the validated Spanish version [21] of the Beck Depression Inventory (BDI-II) [22]. This is one of the most commonly used instruments to assess severity of depressive symptoms in pharmacological and psychological research. This instrument is widely used as it allows patient self-rating of depressive symptoms, avoiding evaluation bias. This is important to our study as, although they will be blinded to treatment condition, it is presumed that it will be easy for evaluators to identify which kind of recommendation each patient received during follow-up assessments. Moreover, the BDI is little influenced by somatic symptoms experienced by patients.

Other variables assessed in our study are:

– Socio-demographic variables such as gender, age, marital status, education level, occupation, economic status and hygienic-dietary habits.

– Psychiatric comorbidity will be assessed by means of the Mini International Neuropsychiatric Interview (MINI) and the State-Trait Anxiety Inventory (STAI). The MINI is a structured diagnostic psychiatric interview designed to diagnose according to DSM-IV and ICD-10 criteria [23,24]. This psychiatric interview will allow a diagnosis of Major Depressive Disorder at the baseline and also will permit the exclusion of other severe comorbid psychiatric disorders. The STAI is a self-rated inventory assessing two independent factors: State anxiety (a transitory emotional condition) and Trait anxiety (relatively stable anxious tendency). This inventory has been adapted and validated in the Spanish population [25].

– Quality of life will be measured with the EuroQol 5D (EQ-5D), which is a standardized instrument used as a measure of health outcome. The Spanish version will be administered [26].

– Health Care Services Consumption will be measured by the Spanish version of the Client Service Receipt Inventory (CSRI) [27], an instrument that allows the collection of data regarding social and sanitary services use as well as other data regarding economic impact (eg: time on sick leave) in the previous twelve months.

Assessments will take place at baseline and after 6 and 12 months.

### Statistical analysis

In the analysis of clinical data, an ‘intention-to-treat’ analysis will be carried out. This analysis will include elemental ‘head-to-head’ descriptions and comparisons between intervention and control groups. Specifically, descriptive statistics (mean and 95% confidence intervals for metric variables with a normal distribution; median and interquartile range for metric variables without a normal distribution; frequency distribution for categorical variables) will be defined for each variable in both groups. To check the main hypothesis all variables will be compared (t0-tk) by means of the ANOVA test with adequate post-hoc contrasts for normally distributed variables or the Kruskal-Wallis H test. Finally, different multivariate analysis including multilevel regression analysis will be included if required. Improvement effect size will be calculated as well.

In the cost-analysis, direct costs will be calculated `ing to the medication-derived costs those derived from the use of sanitary services (visits to Primary Care, Specialized services and Emergencies, as well as hospitalizations). Medical cost will be calculated by determining price per milligram (including VAT) during the study period according to the 2011 Vademecum. Total cost of pharmacological treatment will be calculated multiplying price per milligram by daily dose in milligram and length of treatment (in days). Cost derived from health services and indirect costs will be calculated.

### Ethical aspects

This study has received the approval of the Ethics and Clinical Research Committees of the Balearic Islands. Written informed consent of participants will be obtained before group allocation. Patients will have been properly informed about the study characteristics and objectives previous to study inclusion. They will also be informed about the voluntary nature of participation and the possibility to leave anytime with the guarantee that they will still receive treatment as recommended by their GP. Patients allocated to control group will be offered to follow the intervention group recommendations once the study is finished, if final results recommend doing so. Confidentiality of data will be guaranteed.

## Discussion

This study represents a great opportunity for the improvement with minimal costs of the effectiveness of depression treatment. Results of a preliminary study [9,13] pointed out that significant improvement of depressive symptoms can be achieved with this simple intervention. Its feasibility for the sanitary system derives from its low time consumption for health providers and the low cost of its implementation. Moreover, no significant adverse events or security problems derived from this intervention are expected.

Considering the low-restrictive nature of inclusion and exclusion criteria of the present study, problems for the recruitment of patients are not expected. The sample size will enable the determination of the profile of patients that could benefit from this simple intervention. It will also provide a better understanding of the reasons why some patients do not adhere to these recommendations, with the objective of eliminating this trend. The development of this research line in Primary Health Care settings is of great interest as well.

However, this study also has some limitations that will be taken into account. The main limitation derives from the fact that although GPs will be blinded to patient allocation, it is presumed that they will easily be able to identify which group the patient has been assigned to in the follow-up assessments. This is a potential bias as GPs could be induced to change pharmacological treatments based on this knowledge. In order to minimize this effect, we will ask participant GPs to stay as oblivious to patient allocation as possible. In case patient allocation is suspected, they will be asked to maintain a neutral attitude to patient comments regarding the recommendations they have received. Raters will also be blinded to treatment condition but, again, it will be easy for them to suspect patient allocation from the information provided by patients during the assessments. This could obviously introduce involuntary bias. To correct this possible bias, self-applied instruments will be preferred as main outcome variables.

## Abbreviations

BDI: Beck Depression Inventory; BMI: Body Max Index; BP: Blood Pressure; CSRI: Client Service Receipt Inventory; GP: General Practitioner; HR: Heart Rate; MINI: Mini International Neuropsychiatric Interview; STAI: State-Trait Anxiety Inventory; EuroQol: European Quality of Life self-report questionnaire.

## Competing interests

The authors declare that they have no competing interests.

## Author's contributions

MG is the principal researcher of this project and drafted the manuscript with the assistance of BO and SM. MG, MR, MG, MV, SM, BO, EV and JS were substantially involved in the conception of the study and participated in its design. EV and BO contributed to the development and implementation of the intervention. All authors contributed to the editing of the manuscript and have read and approved the final manuscript.

## Pre-publication history

The pre-publication history for this paper can be accessed here:

http://www.biomedcentral.com/1471-244X/12/201/prepub
